# Coronary magnetic resonance imaging after routine implantation of bioresorbable vascular scaffolds allows non-invasive evaluation of vascular patency

**DOI:** 10.1371/journal.pone.0191413

**Published:** 2018-01-25

**Authors:** Constantin von zur Mühlen, Simon Reiss, Axel J. Krafft, Lisa Besch, Marius Menza, Manfred Zehender, Timo Heidt, Alexander Maier, Thomas Pfannebecker, Andreas Zirlik, Jochen Reinöhl, Peter Stachon, Ingo Hilgendorf, Dennis Wolf, Philipp Diehl, Tobias Wengenmayer, Ingo Ahrens, Christoph Bode, Michael Bock

**Affiliations:** 1 Department of Cardiology and Angiology I, Heart Center Freiburg University, Faculty of Medicine, University of Freiburg, Freiburg, Germany; 2 Department of Radiology–Medical Physics, University Medical Center Freiburg, Freiburg, Germany; 3 ABBOTT Vascular, Wetzlar, Germany; Medstar Washington Hospital Center, UNITED STATES

## Abstract

**Background:**

Evaluation of recurrent angina after percutaneous coronary interventions is challenging. Since bioresorbable vascular scaffolds (BVS) cause no artefacts in magnetic resonance imaging (MRI) due to their polylactate-based backbone, evaluation of vascular patency by MRI might allow for non-invasive assessment and triage of patients with suspected BVS failure.

**Methods:**

Patients with polylactate-based ABSORB-BVS in proximal coronary segments were examined with 3 Tesla MRI directly (baseline) and one year after implantation. For assessment of coronary patency, a high-resolution 3D spoiled gradient echo pulse sequence with fat-saturation, T2-preparation (TE: 40 ms), respiratory and end-diastolic cardiac gating, and a spatial resolution of (1.08 mm)^3^ was positioned parallel to the course of the vessel for bright blood imaging. In addition, a 3D navigator-gated T2-weighted variable flip angle turbo spin echo (TSE) sequence with dual-inversion recovery black-blood preparation and elliptical k-space coverage was applied with a voxel size of (1.14 mm)^3^. For quantitative evaluation lumen diameters of the scaffolded areas were measured in reformatted bright and black blood MR angiography data.

**Results:**

11 patients with implantation of 16 BVS in the proximal coronary segments were included, of which none suffered from major adverse cardiac events during the one year follow up. Vascular patency in all segments implanted with BVS could be reliably assessed by MRI at baseline and after one year, whereas segments with metal stents could not be evaluated due to artefacts. Luminal diameter within the BVS remained constant during the one year period. One patient with atypical angina after BVS implantation was noninvasively evaluated showing a patent vessel, also confirmed by coronary angiography.

**Conclusions:**

Coronary MRI allows contrast-agent free and non-invasive assessment of vascular patency after ABSORB-BVS implantation. This approach might be supportive in the triage and improvement of diagnostic workflows in patients with postinterventional angina and scaffold implantation.

**Trial registration:**

German Register of Clinical Studies DRKS00007456

## Background

Within one year after percutaneous coronary interventions (PCI), up to 30% of patients either re-develop symptoms of angina or continue to suffer from chest pain [1]. This causes a lot of uncertainty in patients but also in physicians, resulting in non-invasive stress tests with often inconclusive results, or invasive coronary angiograms [2]. In the majority of patients, the angiograms confirm a good result at the treated target lesion, and can exclude a progression of coronary artery disease at non-target vessels [3]. Most recent study results on bioresorbable vascular scaffolds (BVS) have also increased uncertainty about the outcome of patients implanted with the polylactate-based scaffold system [4].

Therefore, a non-invasive method for evaluating the results after PCI and stent/scaffold implantation would be desirable. Currently available stent systems, however, can not be evaluated by state-of-the-art imaging techniques. In coronary computed tomography (cCT), metal-based alloys cause artefacts that challenge the assessment of vascular patency and, therefore, stent function [5,6]. In magnetic resonance imaging (MRI), metal also causes artefacts and imaging of mid/distal coronary segments is still challenging due to cardiac motion [7,8]. However, coronary MRI would be preferable as it provides high prognostic value for cardiac events [9] as well as a high negative predictive value for coronary artery disease[10]. Furthermore, MRI enables the assessment of vascular wall structure and functional parameters [11,12,13].

Over the last years, BVS have been developed as an alternative to metal-based stents [14]. Multiple randomized trials have been published during the last years comparing the polylactate-based ABSORB-BVS with drug eluting stents (DES), showing heterogenous results depending on patient/lesion selection and quality of implantation, with increased rates of target vessel revascularization (TVR) and scaffold thrombosis (ST) for ABSORB-BVS [4,15,16]. Since polylactate does not cause metal artefacts, non-invasive evaluation of BVS after implantation would be a helpful option, possibly allowing a triage and improvement of diagnostic workflows in patients with post-interventional angina after BVS implantation. Recently, proof-of-principle of imaging BVS for evaluating vascular patency has been demonstrated [17].

In this study, we therefore performed coronary MRI of patients implanted with the polylactate-based ABSORB-BVS at baseline and after one year in a mixed setting of clinical scenarios.

## Methods

### Ethics approval and consent to participate

The study was approved by the institutional review board (local ethics committee) of the Freiburg University (AZ 120/14), and was registered at the German Register of Clinical Studies (DRKS00007456).

### Patient selection and BVS implantation

The study was approved by the institutional review board (local ethics committee) of the Freiburg University (AZ 120/14), and was registered at the German Register of Clinical Studies (DRKS00007456).

After obtaining written informed consent, patients with a routine implantation of a BVS at our department were included according to the following criteria: implantation of a scaffold within the proximal 45mm of a coronary vessel (for optimal MR image quality); no contraindication against MRI scans (e.g., metal implants); lesion criteria such as defined in the ABSORB II-study [14] (no severe calcification; no ostial lesions; no left main lesions; no bifurcations); no contraindication against 12 months dual antiplatelet therapy; no atrial fibrillation or other significant rhythm disorders; ability to perform a 12-month follow up MRI scan. In this study, we implanted the polylactate-based ABSORB GT1-BVS (ABBOTT Vascular, Wetzlar, Germany), length and diameter was chosen according to the lesion characteristics. For drug eluting stents (DES), we used the SYNERGY everolimus-eluting platinum chromium stent system (BostonScientific, Quincy/MA, USA).

In order to obtain optimal results, BVS were implanted by interventionalists experienced in BVS implantation according to the „5p-rules”(proper vessel sizing; preparation of the lesion by pre-dilatation; pay attention to the expansion limits; post-dilation with NC-ballons; prescribe dual antiplatelet therapy). Radial or femoral access was selected by discretion of the interventionalist.

### Study protocol

Within 7 days after BVS implantation, baseline MRI was performed as described below. Patients were discharged and educated to contact the study center in case of recurrent angina or any other significant cardiac condition requiring re-evaluation of the coronary status. After 11 months, patients were contacted by telephone, and a follow-up scan was scheduled for month 12 (+/- 2 weeks) in the setting of an outpatient visit. At this time point, patients were also interviewed for health status and episodes of recurrent angina.

### Magnetic resonance imaging protocol

Coronary MR imaging was performed on a clinical 3 Tesla MR system (Siemens PRISMA, Erlangen, Germany). Patients were placed on the patient table in supine position, and the system’s ECG system was applied for cardiac triggering. For signal reception, the system’s body (18 channels) and spine (32 channels) array coils were used.

For the assessment of coronary patency, a non-contrast enhanced targeted double-oblique 3D imaging approach was chosen [18]. First, a low-resolution 3D GRE image set covering the whole heart (TE/TR: 1.2/5.2 ms, FoV: 121x248x320 mm^3^, matrix: 22x124x160, voxel size: 5.5x2.0x2.0 mm^3^, R = 2, bandwidth: 445Hz/px, fat-saturation, T2-preparation (TE: 40 ms), respiratory and end-diastolic cardiac gating) was acquired for localization of the culprit vessel. For respiratory gating, an MR navigator signal is used which monitors the breathing motion of the diaphragm in the right lung. The navigator signal is acquired at every heartbeat and when the position of the diaphragm falls within a pre-defined position window. Using the MR system’s three point positioning tool, the imaging slab of a high-resolution 3D GRE pulse sequence was positioned parallel to the course of the vessel. Sequence parameters were set to TE/TR: 1.9 ms/4.1 ms, FoV: 28x277x295 mm^3^, matrix: 26x256x272, voxel size: 1.08x1.08x1.08 mm^3^, parallel imaging acceleration factor: *R* = 2, bandwidth: 635 Hz/px, fat-saturation, T2-preparation (TE: 40 ms), respiratory and end-diastolic cardiac gating.

Then, a 3D navigator-gated T2-weighted variable flip angle TSE sequence with dual-inversion recovery black-blood preparation and elliptical k-space coverage was applied with the following parameters: TE_eff_ = 93 ms, TR = 2*(average duration of cardiac cycle), FoV: 16x234x294 mm^3^, matrix: 14x204x256, voxel size: 1.14x1.15x1.15 mm^3^.

### Image analysis

As the BVS were not directly visible on the re-formatted images, the BVS position in the coronary artery was determined from the X-ray angiograms by measuring the distance of the proximal marker to the next proximal vessel bifurcation or the ostium. 3D MR image data sets were then reformatted along the course of the vessel (IMPAX EE Extended Multi-planar Reconstruction Plugin, AGFA Healthcare), and the BVS position was determined using the X-ray distance. As a measure for image quality, the contrast-to-noise-ratio (CNR) of the scaffolded artery segment was determined as (SI_lumen_-SI_perivascular_)/SD_noise_. Here, SI_lumen_ and SI_perivascular_ are the mean signal intensities of a region of interest (ROI) placed in the scaffolded lumen and the adjacent perivascular area. As parallel imaging was used, the measurement of the true noise is not straight-forward. Here, we measured the apparent noise as the standard deviation SD_noise_ of the signal intensity measured in a ROI placed in the air of the lung in proximity to the heart.

To quantify a potential intraluminal signal decrease caused by the BVS as a result of intravoxel dephasing the luminal SNR of the BVS segment was calculated as SI_lumen_/SD_noise_ and compared to the adjacent segments. The SNR of the adjacent artery segments was measured over a distance of 10 mm on each side of the scaffold and averaged to account for the signal decrease due to decreasing vessel diameters towards the distal end. The averaged SNR of the adjacent segments and the SNR of the BVS segment were then compared using a two-tailed paired Student’s t test.

Potential re-stenosis was assessed by measuring the luminal diameters of the scaffolded vessel segments both at baseline and at 12 months. Furthermore, these diameters were compared to the luminal diameters of the adjacent un-stented segments to evaluate a potential apparent diameter decreases from BVS artifacts. Therefore, the vessel was visualized in a straightened projection of the reformatted image, and the full-width-at-half-of maximum of the signal intensity was calculated along the BVS segment and the segments 10 mm proximal and 10 mm distal from the BVS. This was done with two orthogonal views of the straightened projection to ensure that a potential narrowing of the vessel is also detected if the narrowing is perpendicular to one of the projections. The proximal end of the BVS was localized as described before, and the position of the distal end was then determined using the known length of the BVS. The average diameter of BVS segments and the average diameter of the adjacent segments were calculated for each patient. A two-tailed paired Student’s *t* test was then performed on these values both at baseline and follow up.

The diameters of the BVS segments at baseline and at follow-up were compared based on the ratio of the BVS diameter to the diameter of the adjacent segments which was calculated for each patient at both time points. This was done to compensate for differences in the geometry or imaging artifacts between the baseline and the follow up examination that could affect the apparent overall vessel diameters. The diameters ratios were compared with a two-tailed paired Student’s *t* test.

## Results

### BVS implantation, clinical outcome, 1-year follow up

Implantation of ABSORB-BVS was performed in 11 patients presenting with different clinical scenarios. As depicted in **Table 1**, 9% initially had a stable angina, 27% an unstable angina, 45% a non-ST Elevation myocardial infarction (NSTEMI), and 18% a ST Elevation Myocardial Infarction (STEMI). In all patients, PCI with BVS-implantation was performed successfully without any complications. On average, every patient was implanted with 1.45 scaffolds with a mean diameter of 2.96 ± 0.35mm and a scaffold length of 20.13 ± 5.91mm. According to current standards for BVS-implantation (“5-p-rule”), lesion-preparation and post-dilatation was performed in all of these patients. Baseline MRI was done in average within 1.9 days after PCI, with a 100% success rate, and a mean duration of the complete exam of 49.3 min. Average heart rate during imaging was 70.3 bpm. Black blood imaging was not successful in all patients, as the data was acquired with a long echo train (55 echoes). Hence, in patients with high and/or variable heart rates, an ECG-triggered data acquisition was not possible, such that data acquisition could not be finished or severe motion artifacts were seen in the images. The echo train length was reduced to 45 for the follow-up examinations to increase the number of successful image acquisitions.

**Table 1:** Baseline and procedural characteristics.

Age (years)		63,4 ± 12
Clinical presentation	Stable angina	1 (9%)
	Unstable angina	3 (27%)
	Non-ST-elevation myocardial infarction	5 (45%)
	ST-elevation myocardial infarction	2 (18%)
Cardiovascular risk factors	Diabetes	3 (27%)
	Hypertension	6 (54%)
	Current smoker	4 (36%)
	Hyperlipidemia	1 (9%)
Treated vessel	LAD	7 (63%)
	LCX	3 (27%)
	RCA	4 (36%)
BVS-characteristics	Total scaffolds implanted	16
	Mean Scaffold diameter (mm)	2.96 ± 0.35
	Mean Scaffold length (mm)	20.13 ± 5.91
	Post-Dilation	16/16 (100%)
MRI characteristics	Number of patients with MRI	11/11 (100%)
	Duration (min)	49.3 ± 8.5
	Heart rate (bpm)	70.3 ± 8.1
	Black blood performed	7/11
	Bright blood performed	10/11

In the initial examination of one patient bright blood images could not be acquired due to technical problems with the ECG-signal. However, black blood imaging was successful in this patient.

The 1-year follow up was performed in all 11 patients (**Table 2**). Duration of the MRI exam and heart rate were comparable to baseline MRI (51.5 min, 65.1 bpm). None of the patients suffered from major adverse cardiac events such as myocardial infarction, death, scaffold thrombosis, target lesion revascularization (TLR), or target vessel revascularization (TVR). As discussed below, one patient had a repeat coronary angiogram due to new symptoms of atypical angina, which, however, did not show any progression of coronary artery disease with a good function of the BVS.

**Table 2:** 1-year follow up data.

Patients with one year MRI follow-up	11/11 (100%)
Duration of MRI (min)	51.5 ± 4.5
Heart rate (bpm)	65 ± 6.8
Black blood performed	10/11
Bright blood performed	11/11
Myocardial infarction	0/11 (0%)
Death	0/11 (0%)
Scaffold thrombosis	0/11 (0%)
Target lesion revascularization (TLR)	0/11 (0%)
Target vessel revascularization (TVR)	0/11 (0%)

The average CNR of the bright blood images along the scaffolded segments was 9.8 ± 3.9 at, baseline and 8.6 ± 2.3 at follow up. No significant difference was seen in the luminal SNR between the BVS segment and the adjacent segments both at baseline (p = 0.36) and follow up (p = 0.7). The mean SNR at baseline/follow up was 18.4±5.5/17.9±10.4 for the BVS and 19.0±7.1/18.16±11.3 for the adjacent segments.

**Fig 1:**
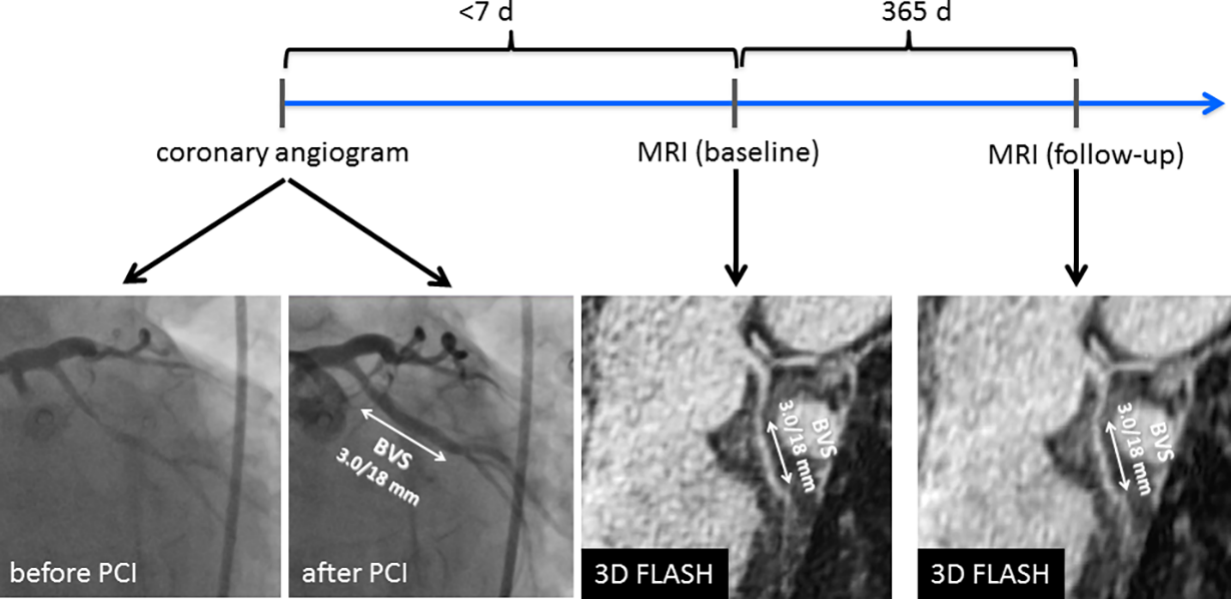
BVS-implantation in the proximal left circumflex artery (LCX). Left: coronary angiogram after implantation of a 3.0/18mm BVS. Middle: MRI at baseline after 2 days, showing the patent scaffolded segment in bright blood (= 3D FLASH) sequences. Follow up one year later confirms the patent vessel in 3D FLASH.

**Fig 1** exemplarily shows a patient with an implantation of a ABSORB-BVS (3.0/18 mm) in the left circumflex artery. At baseline MRI, vessel morphology and the scaffolded vessel were easily recognizable in bright blood sequences. Also in the 1-year follow up, bright blood sequences demonstrate a patent vessel without evidence of restenosis or thrombosis, and the patient was free of symptoms.

**Fig 2:**
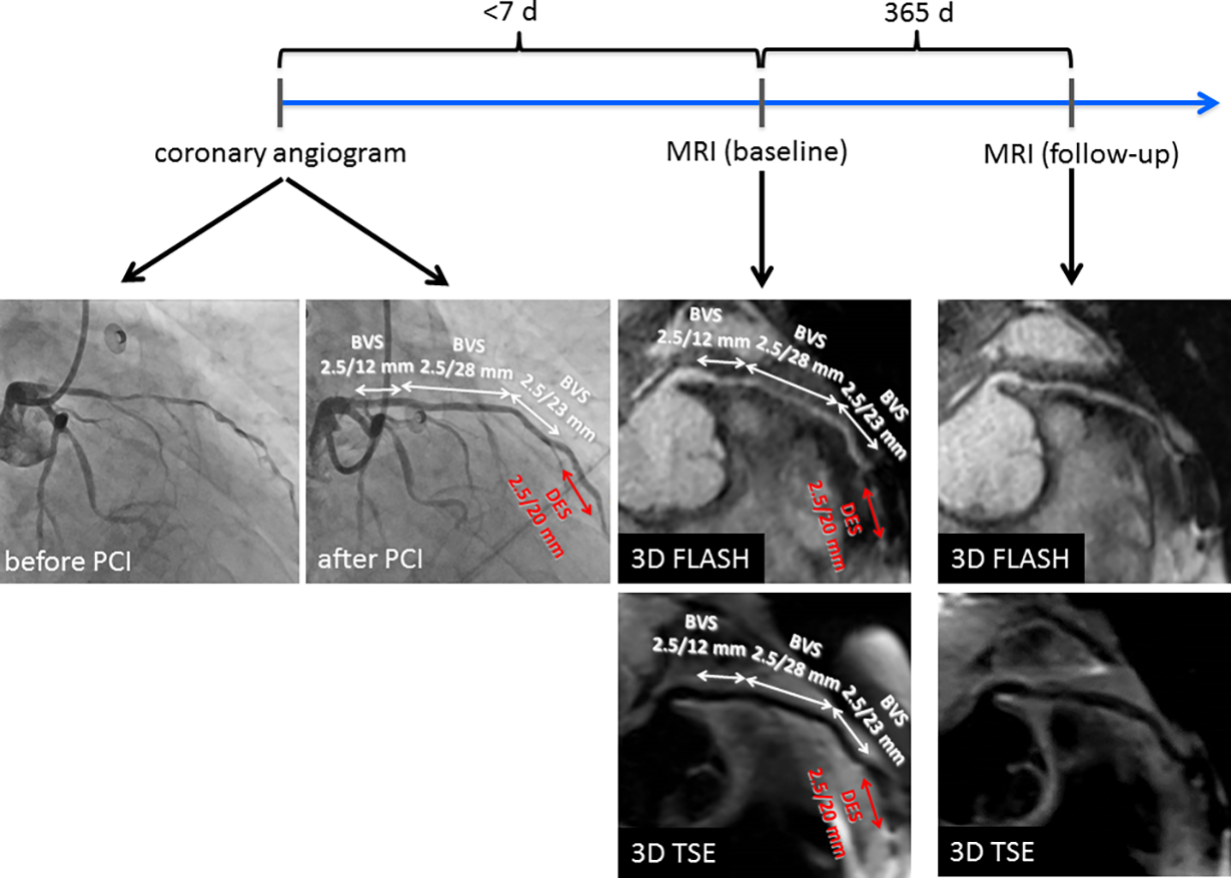
Multiple BVS implantation in the left anterior descending artery (LAD). Left: result in the coronary angiogram with 3 proximal and overlapping scaffolds. One drug eluting stent (SYNERGY 2.5/20mm) was implanted distally, since BVS was not deliverable to this area. MRI at baseline shows open scaffolds in 3D FLASH and 3D TSE, while the distal DES causes significant artefacts not allowing for judgement of vessel patency. Especially the black blood 3D TSE sequences confirm open scaffolds at baseline and after one year in this symptom-free patient.

**Fig 2** demonstrates another patient with diffuse disease of the left anterior descending artery (LAD). Due to multiple significant coronary artery stenoses, 3 ABSORB-BVS were implanted (overlapping scaffold-technique). Due to vessel tortuosity in the mid-LAD segment, which did not allow a BVS delivery, a drug eluting stent (DES) had to be implanted distally to the 3 scaffolds. In baseline MRI, an open vessel is recognizable in bright and black blood sequences. However, in the mid-LAD region with the DES, significant metal artefacts are recognizable, which do not allow for a judgement of vessel patency. Also in the 1-year follow up, black and bright blood sequences show a patent vessel with artefacts in the region of the drug eluting stent; also this patient was free of clinical symptoms.

**Fig 3:**
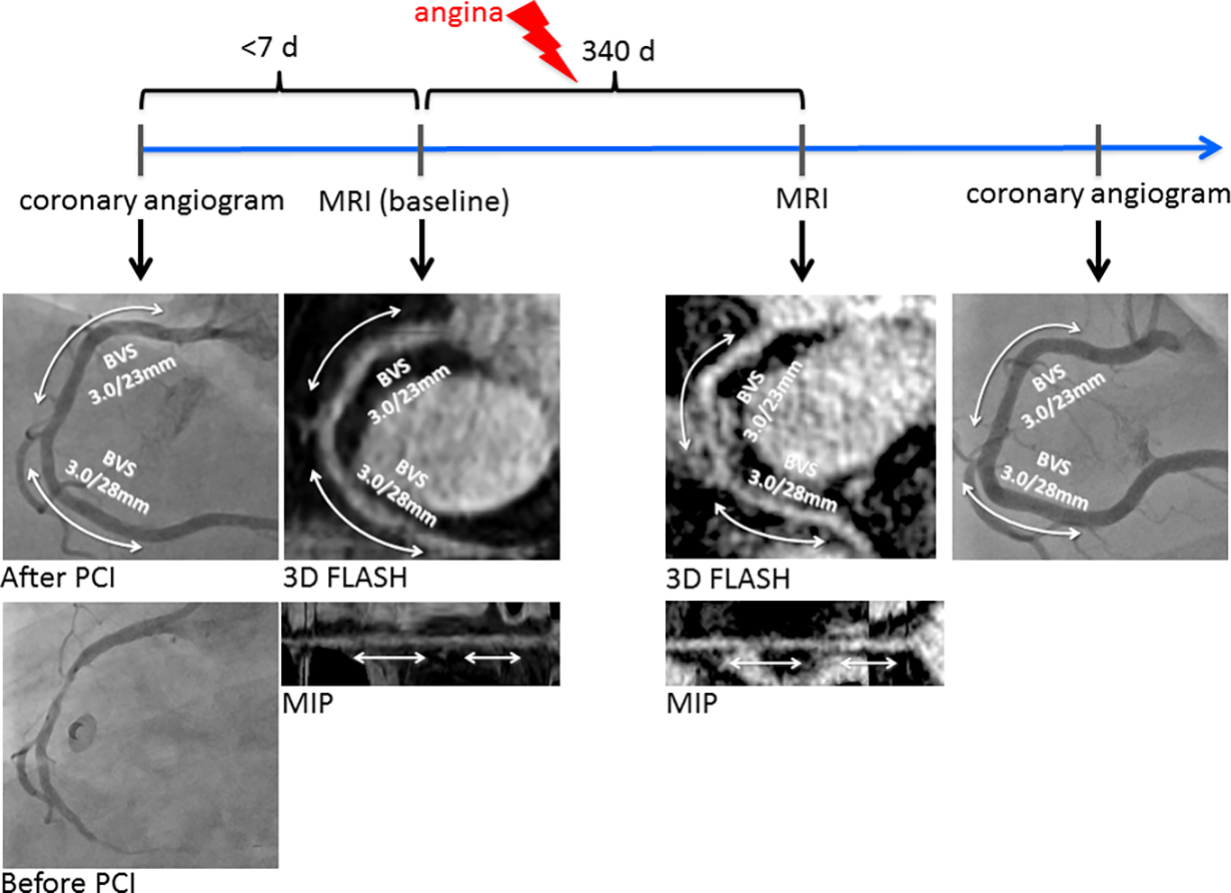
Coronary angiogram and MRI from a patient with two BVS-implantations in the right coronary artery (RCA). Baseline MRI with maximum intensity projection confirms the open scaffold. After 340 days, two weeks before the scheduled 1 year follow-up, the patient presented with atypical chest pain. MRI shows open scaffolds with some artefacts in the coronary segment in between. Coronary angiogram confirmed a good angiographic result.

In **Fig 3**, a patient with 2 ABSORB-BVS in the right coronary artery is shown. Baseline MRI confirms the good result after implantation and demonstrates an open vessel in bright blood images. Unfortunately, black blood imaging could not be performed at baseline; therefore, only bright blood images are shown over the time course. Two weeks before the scheduled 1-year follow up, the patient presented to our emergency department with recurrent symptoms of angina. Since the symptoms were atypical, a treadmill stress test was negative, and there was no sign of myocardial ischemia/necrosis in ECG or laboratory, we decided to perform an MRI exam before conducting another coronary angiogram. In this MRI at 340 days after baseline, bright blood images showed an open vessel especially in the scaffolded regions, although there were some minor artefacts in the segment in between the two scaffolds. A coronary angiogram was performed on the subsequent day and confirmed the MRI results of a very good BVS implantation without progression of coronary artery disease.

As demonstrated in Table 1, at baseline the mean vessel diameter was comparable in the scaffolded and the adjacent segments, showing no significant difference (p = 0.27). After 12 months, the mean diameters showed no significant difference (p = 0.37). In addition, no significant difference was found in the ratio of the BVS diameter and the adjacent diameters at baseline and at follow up p = 0.17).

## Discussion

In this proof-of-principal study, we demonstrate that non-invasive magnetic resonance imaging (MRI) of polylactate-based bioresorbable vascular scaffolds (ABSORB-BVS) is possible in a mixed setting of patients initially presenting with stable angina, unstable angina, NSTEMI, or STEMI. Proximal and mid segments of all coronary vessels with BVS could be imaged either by bright blood or black blood MRI. With bright blood imaging, vascular patency was assessable at baseline and after one year, which might be helpful in the triage of patients presenting with recurrent symptoms of angina after BVS-implantation. None of the patients in this study suffered from major adverse cardiac events, such as device thrombosis, re-stenosis, myocardial infarction, or death.

MRI allowed measuring the dynamics of scaffold diameters over time. Black blood TSE imaging was added to the MR protocol as susceptibility artifacts that could arise from the BVS are reduced in spin echo imaging compared to gradient echo sequences. This study showed that no artifacts are seen with both imaging techniques. Successful black blood imaging with clear delineation of the coronary artery was not possible in all patients which may be attributed to the relatively long acquisition window used for the TSE sequence compared to the bright blood GRE sequence. Bright blood imaging was successful in these patients such that the luminal patency could be assessed and the vessel diameters could be measured in all 11 patients. For further studies, improved imaging techniques such as a recently proposed simultaneous bright and black blood acquisition combined with motion correction may help to increase the success rate of black blood imaging [19]. A general advantage of blood signal attenuation techniques is the improved visualization of the coronary vessel wall compared to bright blood imaging. This helps to identify and quantify plaques as well as to detect positive remodeling [20,21]. This may provide additional insight in cases in which re-stenosis is detected with bright blood imaging.

The difference between the polylactate-based scaffolds and metal-based stents in MRI becomes evident in one of our cases, where both types were implanted. In this case, scaffolded segments can be easily judged without any artefacts. However, the metal-based stent has substantial artefacts caused by susceptibility of metallic wire meshes and radio-frequency screening [22], so that no evaluation of vessel patency was possible. Also the concept of non-invasive triage of patients presenting with new onset of angina after BVS implantation becomes evident in our study: the one patient presenting with atypical angina was easily examinable with coronary MRI, which showed a good result in the areas of BVS implantation.

In this study, we only included patients with lesion criteria corresponding to the ABSORB II-study [14], i.e., we did not include patients with ostial lesions, severe calcification, bifurcation involvement, or left main stenosis. All scaffolds were implanted after 1:1 predilatation with a compliant balloon and were post-dilated with a quarter-size-up non-compliant balloon in 100% of cases, showing an excellent postinterventional result. We did not implant patients with vessel diameters of less than 2.75 mm, which was important for establishing our non-invasive MR imaging protocol.

It may be questionable why MR imaging was used for BVS assessment. The advantage of MRI is that no ionizing radiation is involved, and no contrast agent has to be injected for imaging the coronary vessels. Furthermore, MRI offers the unique opportunity to have a “one stop shop” for morphologic and functional information, such as delayed enhancement, cine-MRI, or endothelial function [23]. Future approaches could also allow for combining this protocol with stress perfusion imaging and measuring coronary flow velocities[24]. Importantly, all MRI sequences in our studies were performed without contrast agent injection.

Recently, also magnesium-based scaffolds have been introduced [25]. The currently available magnesium scaffold (Magmaris, Biotronik, Germany) only contains an overall low amount of magnesium as a metallic element, most likely not causing relevant artefacts. Therefore, the approach of imaging magnesium-based scaffolds might be a promising perspective.

In general, due to the relatively long acquisition times, MR image quality strongly depends on cardiac and respiratory motion, and new techniques will be needed to compensate for motion artefacts, especially in patients with variable heart and breathing rates [18,26,27]. This might also facilitate whole heart imaging for simultaneous functional assessment and high resolution imaging of peripheral coronary arteries.

In this proof of principle study, we only examined 11 patients. However, for obtaining optimal image resolution and quality, strict selection of patients with proximal and mid segment BVS implantation in more or less straight coronary segments was essential in this study, only including patients with no contraindications against repeated MRI scans. Since decision to include the patients into the study was made on the table in the cath lab according to the anatomical and morphological criteria, it was also not possible to perform MRI scans in patients before scaffold implantation: a large number of MRI scans would have been necessary, resulting in only a small number of patients selected for the study in the cath lab. Due to the small number of patients, the study is definitely not powered to evaluate clinical endpoints: target lesion revascularization rate in the ABSORB II-study after one year was 1% [14], which can only evaluated in an MRI study involving a large number of patients in multiple centers. In our patient group, there were no major adverse cardiac events. Definitely, as currently discussed, lesion selection, exact implantation of scaffolds, and post-dilatation is essential for obtaining satisfying long term results [28,29].

An invasive follow up at the one year timepoint would have certainly been of interest as a reference for stent patency, but could not have been performed in the context of this proof of principle study in patients free of symptoms due to ethical issues.

Although desirable, resorption kinetics cannot be reliably assessed by this approach. Spatial image resolution is limited in this setting, which might be overcome after further improvement of the imaging protocol. Although in vitro studies showed that the platinum markers of the scaffold can be recognized by MRI in a phantom setup, the markers are not reliably detectable in vivo, as also described in two other proof-of-principle studies of scaffold imaging [17,30]. Therefore, an orientation towards e.g. side branches is necessary to reliably locate the BVS in the MRI scan. As an alternative, future scaffold generations could be enhanced with iron oxide-based markers at the scaffold ends, which are easier to detect by MRI in dedicated sequences.

Most recent studies have raised concerns in the application of the polylactate-based ABSORB-BVS in clinical routine. In the AIDA-Study, scaffold-thrombosis was significantly increased compared to DES after 2 years (3.5% vs. 0.9%, p<0.001), with no difference in target-vessel failure (TVF) [4]. In the ABSORB-III study comparing the ABSORB-BVS vs. the XIENCE-DES, TLF-rates were significantly increased after 2 years in the patients treated with BVS (10.9% vs. 7.8%, p = 0.03). Adverse events were increased in patients where vessel sizing or implantation was not correctly performed. However, correct lesion selection and implantation techniques with pre- and postdilation of the lesion and the scaffold are crucial, and can result in a significant decrease of TLF and scaffold thrombosis comparable to DES [29]. Due to these most recent study results and a resulting decreased commercial success, the ABSORB-BVS is not available any longer. However, our approach of non-invasive imaging of the ABSORB-BVS might be of interest for those patients presenting with inconclusive clinical symptoms before performing an invasive coronary angiogram.

It is not clear if these data can be transferred to other scaffold systems such the magnesium-based systems, since these have a faster resorption of the system and a different design with potential antithrombotic properties of the material. The data of the non-randomized BIOSOLVE-II/III-study look more promising with lower TLF and no scaffold thrombosis[31], but need confirmation in larger randomized trials.

## Conclusions

In this study, we show that assessment of coronary arteries implanted with a polylactate-based ABSORB-BVS by MRI is possible without the application of contrast agent, allowing evaluation of vascular patency at baseline and follow-up MRI after one year. Furthermore, the difference between the scaffold and metal-based stents in MRI becomes evident, since no material-related artefacts are visible in coronary segments treated with BVS. In patients with recurrent angina after BVS-implantation or inconclusive clinical symptoms, this approach might be helpful to guide triage and diagnostic workflows for evaluating a possible ABSORB-BVS failure.

## Supporting information

S1 Fig Overview of the reformatted bright blood images of all 11 patients acquired at the baseline and follow-up MRI exam. BVS positions are indicated by the arrows.
(TIF)
