# Supplementary figures and images for: Coronary magnetic resonance imaging after routine implantation of bioresorbable vascular scaffolds allows non-invasive evaluation of vascular patency
Source: PLoS One. 2018 Jan 25;13(1):e0191413. doi: 10.1371/journal.pone.0191413 (PMC5784929; doi:10.1371/journal.pone.0191413)

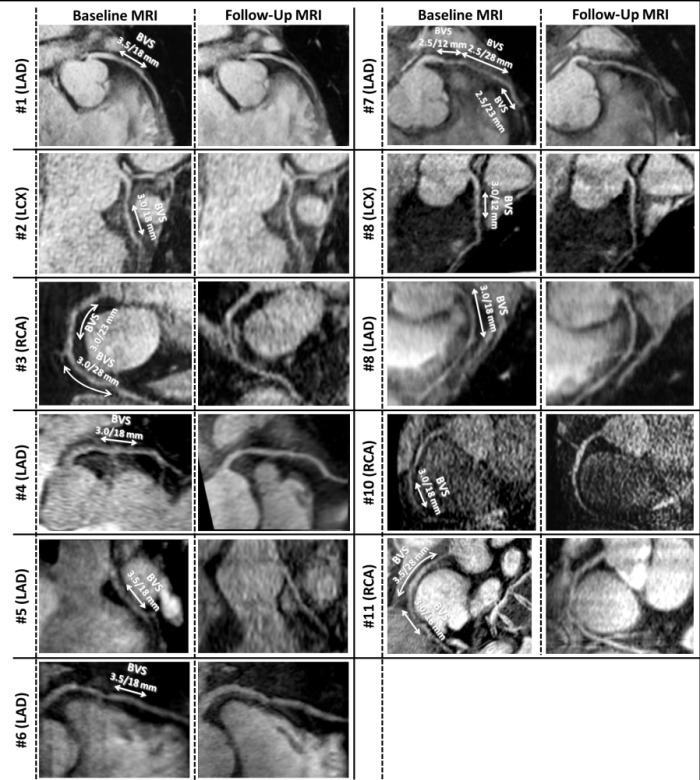

Supplement: S1 Fig — BVS positions are indicated by the arrows. (TIF) [file pone.0191413.s001.tif]
